# The Two Faces of Postural Control in Older Adults: Stability and Function

**DOI:** 10.1016/j.ebiom.2017.03.030

**Published:** 2017-03-25

**Authors:** Ruth Rath, Michael G. Wade

**Affiliations:** Affordance Perception-Action Laboratory, School of Kinesiology, University of Minnesota, USA

Frailty is a geriatric syndrome encompassing a range of biological and behavioral changes that impact our longevity and quality of life, and also highlight a person's vulnerability across multiple forms of stress. In this commentary, we review consequences of the frailty syndrome regarding changes in postural control from two perspectives: First, the physical changes due to declines in a range of physiological systems which lead to an increased incidence of falls in the elderly; second, the functional or ‘embodied’ role of posture as a facilitator of goal oriented activity. The former reviews postural changes as a function of the physiological and biomechanical status of the aging individual, while the latter focuses on changes in the postural system as a facilitator of successful task engagement.

To maintain upright balance, our center of mass (COM) must remain within certain boundaries relative to the feet. Perturbation to stance causes a shift in pressure and can move the COM beyond the “point of no return,” thus causing a loss of balance. Maintenance of posture is a continuously monitored sensorimotor activity that utilizes information from multiple modalities. Any change in equilibrium is perceived and subsequently coupled with an appropriate motoric response in an attempt to maintain stability.

Age-related declines influence the postural control mechanisms which lead to an increased incidence of falls in the elderly. This has been viewed in direct relation to increased motor variability, typically reflected by an increase in the spatial magnitude of body sway. Recent studies of older adults indicate several possible conditions underlying this increased variability. Sarcopenia—a hallmark of the frailty syndrome—results in reduced strength, loss of flexibility, mobility and impaired balance. It has become evident that muscular weakness is the product of multiple neuromuscular changes. Martinikorena et al. (2016) reported a significant relationship between increased variability in gait pattern and the specific deterioration of high-density muscle fibers that support posture; maintaining relative strength preserves muscular robustness, despite a person carrying other markers of frailty. Other studies have reported significant associations between serum vitamin D levels and muscular strength and function, and a significant relationship between low levels of vitamin D and a 4-fold increase in the probability of becoming frail (Sieber, 2017). Thus, it may be important to consider the *quality* of the muscular system when evaluating frailty—such as strength and serum composition—in addition to overall mass.

Recent EMG studies (Kanekar and Aruin, 2014) have established increased onset latency of muscle activation, disruptions in temporal organization, and slower reaction times in response to balance perturbation. Jones et al. (2016) developed a practical EMG method for functional task assessment across relatively short periods of observation, with a near 90% success rate in correctly predicting frailty phenotypes; this is promising, as current clinical recording methods can be cumbersome and often fail to address the *functional* aspect of postural control. Mohler et al. (2016) further expanded this practicality by utilizing wearable technology for detecting significant increases in COM sway among frail older adults living in the community. Such methods can help practitioners assess postural flexibility in older adults necessary to accommodate rapidly changing environmental conditions that often result in a fall.

In addition to the physiological and biomechanical variables associated with frailty, a dynamical systems perspective suggests that postural maintenance across the lifespan encompasses more than simply “maintaining posture for posture's sake.” A growing research literature (for a review see Stoffregen, 2016) concludes that posture, while essential for avoiding falls, plays a key role in support of supra-postural task engagement. This functional role in maintaining control of stance is not autonomous, but integrated or embodied in direct support of a secondary task. Older adults with varying physical and cognitive capacities have demonstrated the ability to self-organize and optimize a postural response for the goal at hand; this inherent flexibility is less to do with age-based manifestations of body sway, and more with the changing biomechanical needs in continued support of postural goals. Age-related decline in function and changes in body morphology necessitate the use of different muscular combinations in support of postural goals; these self-organizing coordinative synergies provide optimized combinations of muscle activation to help maintain equilibrium at any stage of life or physiological status.

The literature suggests that most, if not all, postural responses appear to be in support of a goal. As such, this poses an important question regarding posture as a facilitator of task performance: does this effect change as we age, and if so, how? Munafo et al. (2016) demonstrated that both younger and older adults control their posture in relation to visual targets placed at varying distances, with results indicating reduced positional variability when looking at near targets compared to far targets. With a goal of optimal visual acuity, targets viewed at greater distances required relatively less stability, while closer targets required relatively more stability to produce the same level of acceptable performance; a reduction in postural motion stabilizes the head and eyes, thereby optimizing visual acuity. Stoffregen (2016) made the point that postural control is not just a matter of biomechanical adjustments, but also prioritizes position and motion of the head, which houses the eyes and ears—key receptor systems for balance across the lifespan. The implication here is that posture has a dual role: fall avoidance *and* successful task engagement.

The role of attention in postural control has also been studied in older adults with neurological complications. In a group of older adults with Alzheimer's and Parkinson's disease, Andrade et al. (2014) found that postural control mechanisms were relatively similar during the simultaneous completion of a cognitive task. A significant finding was that *all* subjects—including healthy and diseased—recorded similar postural control performance (relative to baseline) suggesting that older adults prioritize postural maintenance regardless of neurological status. By way of contrast, a study by Jor'dan et al. (2015) employed a visual search task (rather than a cognitive counting task) and reported that Alzheimer's patients could *not* modulate their postural motion. This suggests that a decline in perceptual-action synergy may be an early marker prior to the more memory based signs of dementia. Task specificity would seem to play a role with respect to attentional resources.

Complementary postural therapies have emerged in light of this perspective. A key feature of frailty is reduced functionality of the motor system, which can disrupt the perception-action synergy during task engagement. Wayne et al. (2014) reported that Tai Chi training was a particularly suitable intervention because it integrates balance, flexibility, and neuromuscular coordination along *with* complex attentional processes, such as multi-tasking and focused awareness on sensory modalities. More research is needed on interventions that could provide benefits with respect to postural stability and, perhaps more critically, the role that posture plays as a facilitator of goal oriented behavior. It should be noted that the often invoked phrase “exercise is medicine” is an empirical fact. The value of aerobic activity and progressive resistance training for the elderly — under the guidance of physical therapists and geriatricians — can make significant contributions to maintaining postural stability in the elderly, both in fall avoidance and task engagement.

## Disclosure

The authors declared no conflicts of interest.
